# Short‐term feeding of a ketogenic diet induces more severe hepatic insulin resistance than an obesogenic high‐fat diet

**DOI:** 10.1113/JP275173

**Published:** 2018-08-08

**Authors:** Gerald Grandl, Leon Straub, Carla Rudigier, Myrtha Arnold, Stephan Wueest, Daniel Konrad, Christian Wolfrum

**Affiliations:** ^1^ Institute of Food, Nutrition and Health; ^2^ Physiology and Behavior Laboratory ETH Zürich Schwerzenbach Switzerland; ^3^ Division of Pediatric Endocrinology and Diabetology; ^4^ Children's Research Center University Children's Hospital Zurich Switzerland

**Keywords:** ketogenic diet, high fat diet, insulin resistance

## Abstract

**Key points:**

- A ketogenic diet is known to lead to weight loss and is considered metabolically healthy; however there are conflicting reports on its effect on hepatic insulin sensitivity.
- KD fed animals appear metabolically healthy in the fasted state after 3 days of dietary challenge, whereas obesogenic high‐fat diet (HFD) fed animals show elevated insulin levels.
- A glucose challenge reveals that both KD and HFD fed animals are glucose intolerant.
- Glucose intolerance correlates with increased lipid oxidation and lower respiratory exchange ratio (RER); however, all animals respond to glucose injection with an increase in RER.
- Hyperinsulinaemic–euglycaemic clamps with double tracer show that the effect of KD is a result of hepatic insulin resistance and increased glucose output but not impaired glucose clearance or tissue glucose uptake in other tissues.

**Abstract:**

Despite being a relevant healthcare issue and heavily investigated, the aetiology of type 2 diabetes (T2D) is still incompletely understood. It is well established that increased endogenous glucose production (EGP) leads to a progressive increase in glucose levels, causing insulin resistance and eventual loss of glucose homeostasis. The consumption of high carbohydrate, high‐fat, western style diet (HFD) is linked to the development of T2D and obesity, whereas the consumption of a low carbohydrate, high‐fat, ketogenic diet (KD) is considered healthy. However, several days of carbohydrate restriction are known to cause selective hepatic insulin resistance. In the present study, we compare the effects of short‐term HFD and KD feeding on glucose homeostasis in mice. We show that, even though KD fed animals appear to be healthy in the fasted state, they exhibit decreased glucose tolerance to a greater extent than HFD fed animals. Furthermore, we show that this effect originates from blunted suppression of hepatic glucose production by insulin, rather than impaired glucose clearance and tissue glucose uptake. These data suggest that the early effects of HFD consumption on EGP may be part of a normal physiological response to increased lipid intake and oxidation, and that systemic insulin resistance results from the addition of dietary glucose to EGP‐derived glucose.

## Key points

- A ketogenic diet is known to lead to weight loss and is considered metabolically healthy; however there are conflicting reports on its effect on hepatic insulin sensitivity.
- KD fed animals appear metabolically healthy in the fasted state after 3 days of dietary challenge, whereas obesogenic high‐fat diet (HFD) fed animals show elevated insulin levels.
- A glucose challenge reveals that both KD and HFD fed animals are glucose intolerant.
- Glucose intolerance correlates with increased lipid oxidation and lower respiratory exchange ratio (RER); however, all animals respond to glucose injection with an increase in RER.
- Hyperinsulinaemic–euglycaemic clamps with double tracer show that the effect of KD is a result of hepatic insulin resistance and increased glucose output but not impaired glucose clearance or tissue glucose uptake in other tissues.

## Introduction

Despite being one of the pressing healthcare challenges of our time, the aetiology of type 2 diabetes (T2D) is still incompletely understood. Although the proximate cause of T2D is the failure of the pancreatic islet to increase or maintain insulin production, the ultimate cause has not yet been conclusively identified. Overall, there is a link between an unhealthy Western diet and a lack of physical activity, and the development of T2D (van Dam *et al*. 2002). In terms of the progression of the disease, T2D manifests as a slow and worsening decrease of insulin sensitivity affecting multiple organs, during which time blood glucose levels are maintained within normal levels, whereas insulin levels rise to compensate, followed by the eventual failure of the pancreatic islet. Several groups report impairment of systemic glucose tolerance, as well as insulin signalling, in the brain or liver after only a very few days of feeding an obesogenic high‐fat diet (HFD) to rodents, with the diet typically consisting of 60% of calories from fat and ∼20% each from protein and carbohydrates (Wang *et al*. 2001; Ono *et al*. 2008; Clegg *et al*. 2011; Wiedemann *et al*. 2013). These early impairments of glucose homeostasis typically present as a failure of elevated glucose or insulin levels to supress hepatic glucose output, leading to decreased glucose tolerance. The development of profound insulin resistance in the major metabolic organs muscle and adipose tissue takes longer and has been linked to inflammation (Rask‐Madsen & Kahn, 2012). By contrast, the manifestation of systemic glucose intolerance, as a consequence of starvation or the ingestion of very low carbohydrate, ketogenic diets (KD) for several days, has been recognized for at least a century in animals and humans and is termed starvation diabetes (Peters, 1945; Lundbaek, 1948). Starvation diabetes was shown to be linked to an increase in hepatic gluconeogenesis by tracer studies in humans (Fery *et al*. 1990), yet any mention of the term starvation diabetes or the citation of related studies is absent from the recent literature concerning the effects of longer‐term KD feeding on glucose tolerance and insulin sensitivity. Even though the efficacy of chronic KD feeding with respect to causing weight loss is accepted (Rosenheck *et al*. 2005; Nordmann *et al*. 2006; Hession *et al*. 2009; Santos *et al*. 2012; Paoli *et al*. 2013), there is significant controversy about its effect on glucose tolerance and insulin sensitivity. Kennedy *et al*. (2007) showed that KD improved glucose tolerance and insulin signalling in mice compared to mice maintained on a HFD for 12 weeks. Badman *et al*. (2009) showed that KD increases glucose sensitivity in *ob/ob* mice independently of weight loss. On the other hand, Jornayvaz *et al*. (2010) showed that, in C57BL/6 mice, 5 weeks on a KD induced hepatic insulin resistance, decreased glucose tolerance, and increased gluconeogenesis, despite preventing weight gain and increasing energy expenditure. Furthermore, Garbow *et al*. (2011) found increased hepatic steatosis and inflammation, as well as decreased glucose tolerance, despite high muscle and adipose tissue insulin sensitivity, in mice on a KD for 12 weeks. Given the similar reported effects on hepatic gluconeogenesis in starvation diabetes and mice maintained KD for several weeks, as well as reported metabolic abnormalities of mice fed an obesogenic HFD for several days, we aimed to address the controversy surrounding KD feeding by analysing the effects of short‐term feeding on glucose tolerance and insulin sensitivity and comparing it with obesogenic HFD.

## Methods

### Ethical approval

All mouse experiments described in the present study were carried out in strict accordance with the recommendations in the Animal Welfare Ordinance (TSchV 455.1) of the Swiss Federal Food Safety and Veterinary Office. The study was approved by the Zurich Cantonal Veterinary Office, Switzerland. Animals were administered buprenorphin (Temgesic; 50–100 μg kg^–1^) for analgesia prior to surgery and were anaesthetized during surgery with isoflurane (4–5% for induction, 1.5–2.5% for maintenance). Post‐surgery animals were injected with glucosaline (glucose in physiological 0.9% saline) (300 μL) and 5 mg kg^–1^ carprofen daily. Animals were killed by cervical dislocation or CO_2_ asphyxiation in slowly rising levels of CO_2_. The investigators understand the ethical principles under which *The Journal of Physiology* operates and confirm that their work complies with the animal ethics checklist outlined in Grundy (2015).

### Animals

C57BL/6 mice were housed in a pathogen‐free animal facility under a 12:12 h light/dark cycle (lights off 07.00 h) at an ambient temperature of 23°C with free access to food and water. Mice were obtained from Charles River (Wilmington, MA, USA) at ∼6–8 weeks old and housed in the animal facility until beginning of the experiment at 12 weeks of age. Mice were fed standard chow (purified diet #2222; Kliba‐Nafag, Kaiseraugst, Switzerland; 18% protein, 7% fat, 58% carbohydrate by mass), a ketogenic diet with 90% of calories derived from fat (Teklan TD.96355; Envigo, Huntingdon, UK; 15.3% protein, 67.4% fat, 0.6% carbohydrate by mass), or a 60% calories high‐fat diet (purified diet #2127; Kliba‐Nafag; 23.9% protein, 35% fat, 23.2% carbohydrate by mass). For the present study 149 mice were killed.

### Plasma collection

Blood was collected from the tail vein into tubes containing 0.5 m EDTA and centrifuged to derive plasma. For post mortem blood collection, animals were killed by CO_2_ asphyxiation and blood was taken by heart puncture.

### Plasma analysis

Blood glucose was measured using an Aviva Accu‐Chek glucose strip system (#07400918016, #06453988016; Roche Diagnostics International, Basel, Switzerland), taking ∼0.6 μL of blood per measurement. Insulin was measured using an MSD enzyme‐linked immunosorbent assay kit (K152BZC; Meso Scale Diagnostics, Rockville, MD, USA). Free fatty acids were measured using the Wako Nefa kit #9196 (Wako Pure Chemical Industries, Tokyo, Japan), triglycerides (TG) were measured with the Cobas Roche/Hitachi Kit #11489232 (Roche Diagnostics International) and cholesterol was measured using the Cobas Roche/Hitachi Kit #11877771 (Roche Diagnostics International). Total ketone bodies were determined using the Wako Chemicals Kit #415‐73301/411‐73401 (Wako Pure Chemical Industries).

### Glucose/insulin tolerance test

Animals were fasted at 08.00 h in the morning and injected i.p. with 1.5 g kg^–1^ body weight of glucose [glucose tolerance test (GTT)] or 0.5 U kg^–1^ body weight of insulin [insulin tolerance test (ITT)] in 0.9% saline after a 6 h fast. Blood samples were obtained from tail tip bleedings for blood glucose measurements at before injection and at 15, 30, 60, 90 and 120 min after injection.

### Indirect calorimetry

Indirect calorimetry was performed with a metabolic cage system (PhenoMaster; TSE systems, Bad Homburg, Germany). On the basis of sequential measurement points (sample interval 20 min) O_2_ consumption (${\dot{V}}_{O_{2}}$) and CO_2_ production (${\dot{V}}_{CO_{2}}$) were calculated using the manufacturer's software (TSE PhenoMaster, version 5.6.5) with the coefficients of 3.941 ($C{\dot{V}}_{O_{2}}$) and 1.106 ($C{\dot{V}}_{CO_{2}}$) and normalization to the lean body mass. Respiratory exchange ratio (RER) was measured as the ratio of CO_2_ produced over O_2_ consumed. Mice were acclimated to the system before measurements. At the beginning of the experiment, mice were switched to HFD or KD, or left on chow. Water and food was available *ad libitum* during the whole measurement, and food weight was measured daily. A multidimensional infrared beam system assessed the locomotor activity which was defined as the total number of beam breaks in the *x*‐ and *y*‐axis. Room temperature was set to 23°C; the light/dark cycle in the room was 12:12 h (lights off at 07.00 h). Mice were monitored for a total of 3 days. Lean body mass of each animal was determined by echo magnetic resonance imaging before and after the experiment.

### Glucose clamp studies

Hyperinsulinaemic–euglycaemic clamp studies were performed in freely moving mice as described previously (Wueest *et al*. 2010). Mice were anesthetized with isoflurane and given analgesia (buprenorphine, 0.1 mg kg^–1^) and eye ointment was applied to both eyes (vitamin A; Bausch & Lomb Swiss AG, Switzerland). A polyurethane catheter was inserted into the right jugular vein and exteriorized at the neck. Post‐surgery, mice were given daily analgesia with carprofen (nororcarp, 5 mg kg^–1^) for 3 days. Five to 7 days after the surgery, glucose clamp was performed in mice that had lost less than 10% of their pre‐operative weight. Mice were fasted at 08.00 h in the morning. Basal glucose production was measured after 4 h 30 min for 80 min. For the clamp, insulin was infused at a constant rate (18 and 12 mU kg^–1^ min^–1^) and steady‐state glucose infusion rate was calculated once glucose infusion reached a more or less constant rate for 15–20 min with blood glucose levels at 4–5 mm (and 6–7 mm, respectively). Glucose concentration was determined using Aviva Accu‐Chek strips every 5 min by sampling blood from a small incision in the tail tip. The glucose disposal rate was calculated by dividing the rate of [3‐^3^H] glucose infusion by the plasma [3‐^3^H] glucose specific activity at steady‐state. Endogenous glucose production during the clamp was calculated by subtracting the glucose infusion rate from the glucose disposal rate. To assess tissue‐specific glucose uptake, a bolus (10 μCi) of 2‐[1‐^14^C]deoxyglucose was administered via catheter at the end of the steady‐state period. Blood was sampled 2, 15, 25 and 35 min after bolus delivery. The area under the curve of disappearing plasma 2‐[1‐^14^C] deoxyglucose was used together with tissue concentration of phosphorylated 2‐[1‐^14^C] deoxyglucose to calculate glucose uptake as described previously (Chin *et al*. 2015).

### Statistical analysis

All data are reported as the mean ± SEM. Statistical analysis was performed using Prism (GraphPad Software Inc., San Diego, CA, USA). Correlation was analysed by calculating the Pearson correlation coefficient. Differences between groups were analysed by ANOVA with a Bonferroni *post hoc* test. *P* ≤ 0.05 was considered statistically significant.

## Results

### Short‐term obesogenic HFD feeding but not KD causes an increase in fasting insulin and homeostatic model assessment‐insulin resistance (HOMA‐IR)

**Figure 1:**
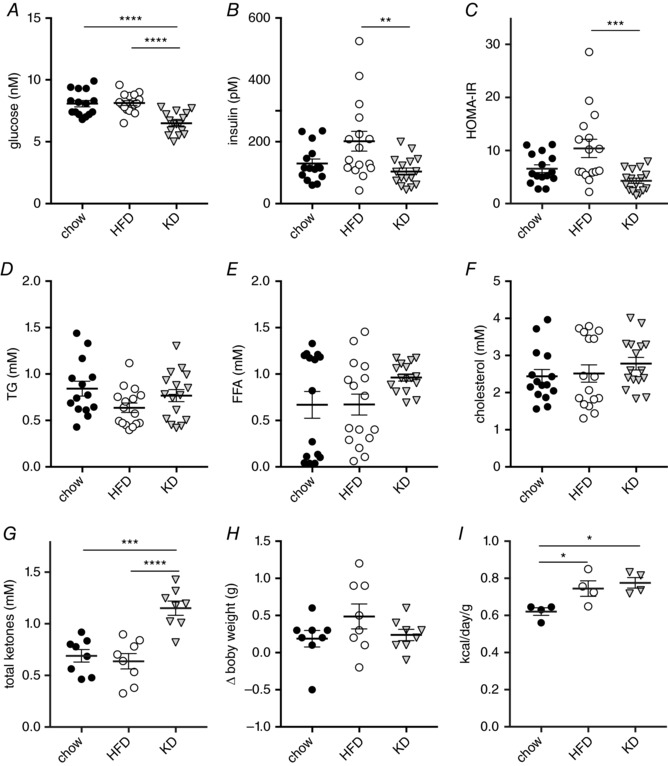
Short‐term obesogenic HFD feeding but not KD causes increases in fasting insulin and HOMA‐IR Plasma metabolic parameters of chow *vs*. 3 days of KD or HFD‐fed mice after a 6 h fast. *A*, glucose levels. *B*, plasma insulin. *C*, calculated homeostatic model assessment of insulin resistance HOMA‐IR. *D*, plasma TG. *E*, plasma FFA. *F*, plasma cholesterol. *G*, plasma total ketones. *H*, body weight change after 3 days of diet. *I*, average energy intake (*n* = 4–16). Data are plotted as the mean ± SEM. ^*^ *P* < 0.05, ^**^ *P* < 0.01, ^***^ *P* < 0.001, ^****^ *P* < 0.0001 by ANOVA.

First, we assessed metabolic parameters in unchallenged mice after a 6 h fast, comparing mice fed a standard chow diet with mice fed a 60% HFD, or a low carbohydrate KD for 3 days. We found that, after 3 days of KD feeding, plasma glucose was slightly reduced compared to chow or HFD (Fig. 1 *A*), whereas plasma insulin levels (Fig. 1 *B*) were not significantly different after a 6 h fast, although they were elevated in HFD fed mice compared to KD. The use of these parameters to calculate the HOMA‐IR metric to quantify insulin resistance suggests that KD fed mice are slightly more insulin sensitive than chow fed mice after 3 days, whereas HFD fed mice show slightly impaired insulin signalling according to HOMA‐IR compared to KD mice (Fig. 1 *C*). Because HOMA‐IR is based only on a correlation between fasted glucose and insulin levels, it does not necessarily reflect actual glucose tolerance. Because fasting plasma free fatty acid (FFA) levels have been reported to correlate strongly with insulin resistance, we measured FFA after a 6 h fast. We found a slight but not significant increase in plasma FFA in the fasted state for KD *vs*. chow. Furthermore, we found no difference in TG (Fig. 1 *E*) or cholesterol (Fig. 1 *F*) between chow and KD fed animals after a 6 h fast, and only small differences between HFD and chow fed animals. As expected from the diet, total plasma ketones were significantly increased in the animals on KD compared to chow and HFD fed animals (Fig. 1 *G*). After 3 days of the respective diets, no significant difference in body weight gain was yet apparent (Fig. 1 *H*), despite an increased caloric intake on HFD and KD, which both show an increased caloric density compared to chow (Fig. 1 *I*). These data indicate that, in the basal state, 3 days of a KD appears to have a neutral to beneficial effect on insulin sensitivity, suggesting a healthy, glucose tolerant state.

### Short‐term KD or HFD feeding causes impaired glucose clearance and insulin tolerance

**Figure 2:**
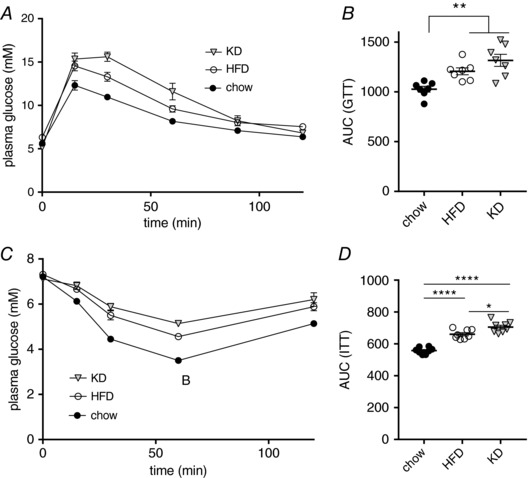
Short‐term KD or HFD feeding causes impaired glucose clearance and insulin tolerance *A*, i.p. GTT of mice fed chow, KD or HFD for 3 days, after a 6 h fast. *B*, AUC of GTT. *C*, i.p. ITT of mice fed chow, KD or HFD for 3 days, after a 6 h fast. *D*, AUC of ITT (*n* = 7–8). Data are plotted as the mean ± SEM. ^*^ *P* < 0.05, ^**^ *P* < 0.01, ^****^ *P* < 0.0001 by ANOVA.

To assess glucose sensitivity, we next challenged animals with an i.p. GTT after 3 days of a HFD or KD diet (Fig. 2 *A* and *B*). Unlike our findings in the basal fasted state, we found a marked and significant decrease in glucose clearance of both HFD and KD fed animals compared to chow fed animals. We also performed an i.p. ITT after 3 days of diet (Fig. 2 *C*). The response in the ITT showed a similar impairment as in the GTT (Fig. 2 *D*). These data suggest that, despite an apparently improved insulin sensitivity in the fasted state, 3 days of KD feeding is sufficient to cause impaired glucose homeostasis in C57/Bl6 mice, comparable to the known adverse short‐term effects of a HFD feeding regime.

### Steady‐state RER inversely correlates with glucose tolerance, although all groups respond to an i.p. glucose challenge with an increase in RER

**Figure 3:**
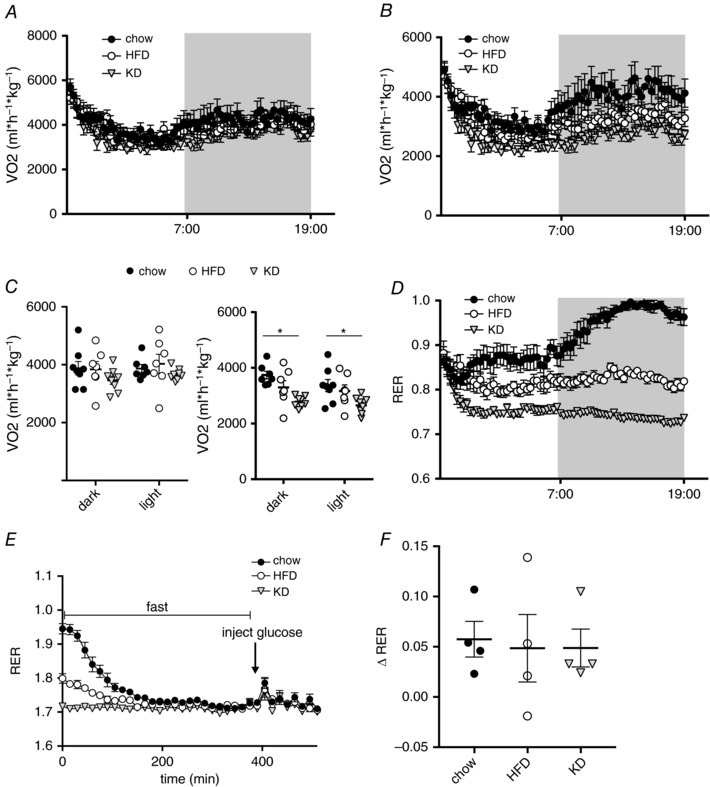
Steady‐state RER inversely correlates with glucose tolerance, although all groups respond to an i.p. glucose challenge with an increase in RER *A*, ${\dot{V}}_{O_{2}}$ used during the first 24 h after switching animals from chow to KD or HFD. *B*, ${\dot{V}}_{CO_{2}}$ produced during the first 24 h after switching animals from chow to KD or HFD. *C*, average ${\dot{V}}_{O_{2}}$ and ${\dot{V}}_{CO_{2}}$ during light and dark phase in the first 24 h after diet switch. *D*, RER of mice fed KD, HFD or chow during the first 24 h. *E*, RER during GTT on day 3; arrow indicates the time of glucose injection after a 6 h fast. *F*, change in RER directly after i.p. injection of glucose (*n* = 4–8). Data are plotted as the mean ± SEM. ^*^ *P* < 0.05 by ANOVA.

The feeding of a KD for 3 days is metabolically similar to the interventions leading to the observed effect of starvation diabetes, and the suggestion has been made that a decreased ability to oxidize glucose, resulting from metabolic adaptation to lipids, might be at least partly responsible for the effect (Anderson & Herman, 1972). Therefore, we aimed to measure systemic substrate usage by measuring ${\dot{V}}_{O_{2}}$ and ${\dot{V}}_{CO_{2}}$ by indirect calorimetry. We found no differences between the three groups in ${\dot{V}}_{O_{2}}$ consumed normalized to lean body mass (Fig. 3 *A* and *C*), suggesting that reported differences in energy expenditure between these different feeding regimens (Jornayvaz *et al*. 2010; Hatori *et al*. 2012) only occur after longer periods of feeding. We did find differences in ${\dot{V}}_{CO_{2}}$, as might be expected by shifting substrate usage for oxidative phosphorylation from carbohydrates to fat (Fig. 3 *B* and *C*). These changes in ${\dot{V}}_{CO_{2}}$ caused marked differences in the RER. Measuring the RER during the diet switch showed that, after ∼16 h, the animals arrived at their respective steady‐state RERs, with the KD fed animals reaching an RER of ∼0.7, indicating almost complete reliance on lipids as a fuel, whereas the HFD fed group reached an RER of ∼0.8 and the RER of the chow fed group remained close to 1.0 (Fig. 3 *D*). Interestingly, these steady‐state RERs correlated very well with the observed glucose intolerance and insulin resistance in the GTT and ITT, respectively (Fig. 2). We obtained a Pearson correlation coefficient of *r* = –0.9991, *R* ^2^ = 0.9995 and *P* = 0.0146 for RER *vs*. AUC_GTT_ and a Pearson correlation coefficient of *r* = –0.9981, *R* ^2^ = 0.9961 and *P* = 0.039 for RER *vs*. AUC_ITT_. One possible explanation occasionally evoked to explain the effects of starvation diabetes is that the metabolic shift to lipids causes impairments in the ability to metabolize or take up glucose and our observation concerning the correlations of RER with glucose clearance and insulin sensitivity appears to support such a hypothesis. To test this, we performed a GTT on animals during continuous indirect calorimetry measurements. We found that all groups immediately reacted to the i.p. injection of glucose after a 6 h fast with a brief but marked increase in RER (Fig. 3 *D* and *E*). This suggests that the ability and propensity to metabolize glucose is unaltered, despite a marked metabolic shift in the primary oxidation fuel caused by adaptation to the various diets, and that the observed changes in glucose homeostasis are not directly a result of the change in the primary metabolic substrate.

### Hyperinsulinaemic–euglycaemic clamps reveal differences in insulin‐suppressed endogenous glucose production but not glucose disposal or tissue glucose uptake

**Figure 4:**
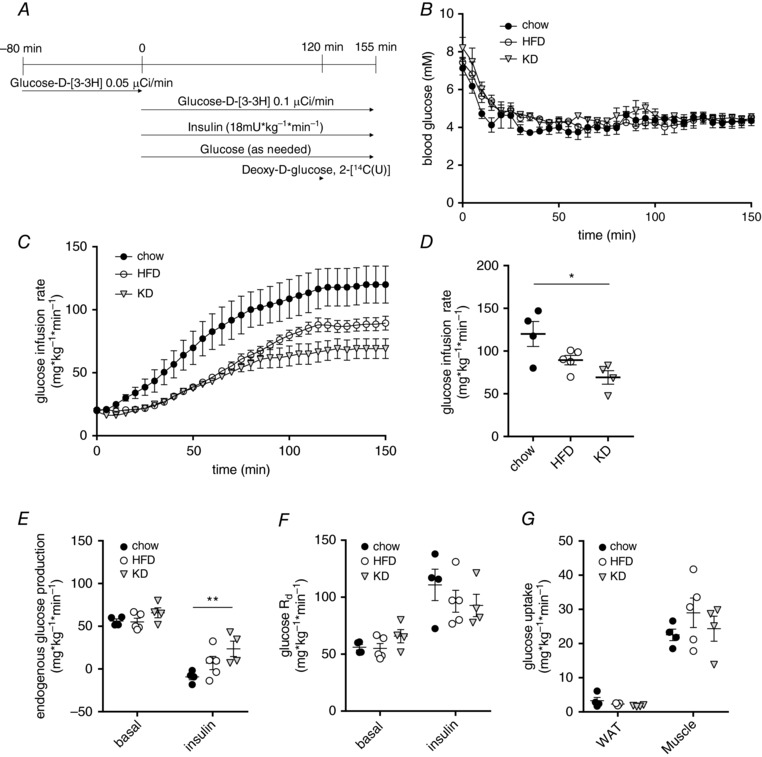
Hyperinsulinaemic–euglycaemic clamps reveal differences in insulin‐suppressed endogenous glucose production but not tissue glucose uptake *A*, scheme of glucose clamp procedure. *B*, blood glucose levels during clamp. *C*, glucose infusion rates during clamp. *D*, glucose infusion rates at steady‐state. *E*, rates of basal and insulin‐inhibited endogenous glucose production. *F*, rates of basal and insulin‐stimulated glucose disappearance (R_d_). *G*, glucose uptake during hyperinsulinaemic–euglycaemic clamps into epididymal WAT and skeletal muscle (quadriceps) (*n* = 4–5). Data are plotted as the mean ± SEM. ^*^ *P* < 0.05, ^**^ *P* < 0.01 by ANOVA.

To assess whole body and tissue‐specific insulin sensitivity in mice fed the three different diets, hyperinsulinaemic–euglycaemic clamps were performed. We used [3‐^3^H] labelled glucose to estimate basal and insulin‐suppressed endogenous glucose production (EGP; mainly reflecting hepatic glucose production) and a bolus of 2‐[1‐^14^C] labelled deoxyglucose was given after reaching steady‐state glucose infusion to measure tissue‐specific glucose uptake. We infused 18 mU kg^–1^ min^–1^ insulin and clamped the mice at a plasma glucose level of 5 mm (Fig. 4 *A*). The glucose infusion rate necessary to achieve euglycemia (Fig. 4 *B*) was significantly lower in mice after 3 days of KD feeding compared to chow fed mice, whereas values for the HFD‐fed mice were between those for KD and chow (Fig. 4 *C* and *D*). This situation is somewhat similar to that observed during the IPGTT (Fig. 2 *A*), where the temporal dynamics showed a slightly better glucose clearance of HFD animals compared to KD, whereas both groups were clearly impaired in glucose tolerance compared to chow. Although basal EGP was similar between the groups, the ability of insulin to supress EGP was significantly reduced in KD compared to chow‐fed mice (Fig. 4 *E*). Conversely, neither the rates of systemic glucose disappearance (Fig. 4 *F*), nor glucose uptake into white adipose tissue (WAT) and muscle (Fig. 4 *G*) were changed. These data indicate that reduced insulin sensitivity observed in KD fed mice is mainly mediated by blunted hepatic insulin sensitivity.

### Hyperinsulinaemic–euglycaemic clamps with lower insulin and glucose targets confirm effect of reduced insulin‐suppressed endogenous glucose production

**Figure 5:**
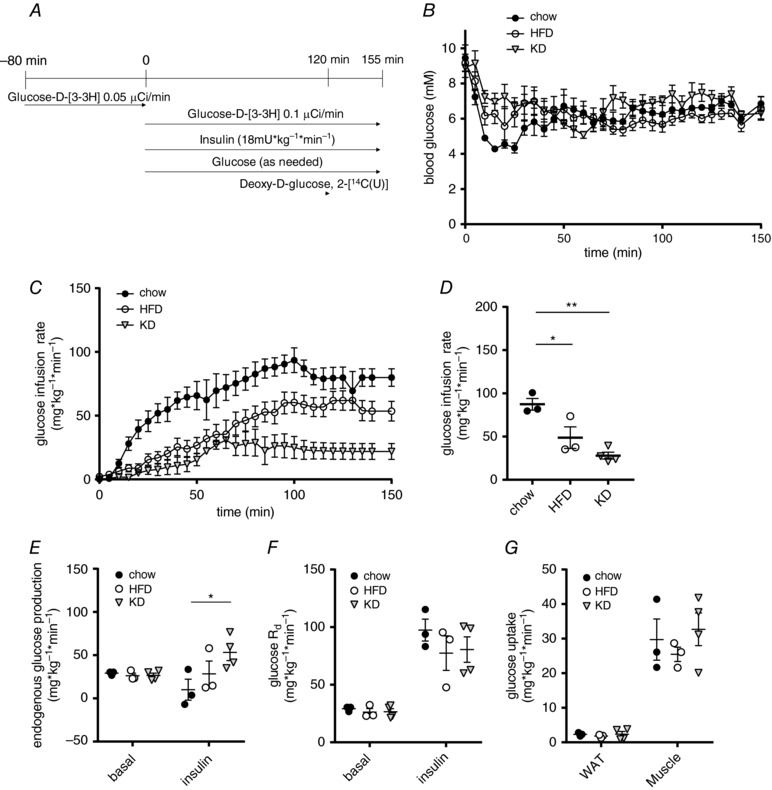
Repeating hyperinsulinaemic–euglycaemic clamps with lower insulin and glucose targets confirms an effect via insulin‐suppressed endogenous glucose production *A*, scheme of glucose clamp procedure. *B*, blood glucose levels during clamp. *C*, glucose infusion rates during clamp. *D*, glucose infusion rates at steady state. *E*, rates of basal and insulin‐inhibited endogenous glucose production. *F*, rates of basal and insulin‐stimulated glucose disappearance (*R*_d_). *G*, glucose uptake during hyperinsulinaemic–euglycaemic clamps into epididymal WAT and skeletal muscle (quadriceps) (*n* = 3–4). Data are plotted as the mean ± SEM. ^*^ *P* < 0.05, ^**^ *P* < 0.01 by ANOVA.

To further confirm these findings, we repeated the hyperinsulinaemic–euglycaemic clamp studies with lower infusion rates of insulin (12 mU kg^–1^ min^–1^) and higher plasma glucose targets (6–7 mm), using [3‐^3^H] labelled glucose to estimate basal and insulin‐suppressed endogenous glucose production and a bolus of 2‐[1‐^14^C] at steady‐state to measure tissue glucose uptake (Fig. 5 *A*). In this setting, HFD and KD fed animals needed significantly lower rates of glucose infusion to achieve plasma levels in the target range (Fig. 5 *B*–*D*). Again, we did not observe a difference in basal EGP, although there was a significantly increased EGP of KD animals in the insulin suppressed state (Fig. 5 *E*). Similar to the clamps with higher insulin infusion and lower glucose targets, there was no significant difference in the systemic rate of glucose disappearance (Fig. 5 *F*) or WAT and muscle glucose uptake (Fig. 5 *E*). These data confirm our finding that the reduced insulin sensitivity observed in KD fed mice is mainly mediated by blunted hepatic insulin sensitivity affecting glucose output, across different ranges of insulin infusion and target plasma glucose levels.

## Discussion

Given the controversy regarding the link between KD and insulin sensitivity, we analysed glucose tolerance in response to short‐term dietary challenges of KD and HFD *vs*. a chow diet. The results of the present study demonstrate that, in the context of *ad libitum* feeding of both HFD or KD for a short period, the effect on systemic glucose tolerance is a result of the inability of insulin to suppress hepatic glucose output, whereas muscle and adipose tissue glucose uptake are completely unperturbed. In our initial hyperinsulinaemic–euglycaemic clamp study, we chose rather high insulin levels during the clamp (18 mU kg^–1^ min^–1^), leading to a complete suppression of hepatic glucose output under insulin and even negative values for EGP in some cases. We aimed to address this by repeating the clamp studies with lower insulin levels (12 mU kg^–1^ min^–1^) and higher glucose targets. Although we were able to confirm our initial findings, it should be noted that our insulin levels still are high and lead to almost complete suppression of HGP.

In longer‐term studies, 5 weeks of KD feeding resulted in a similar phenotype to the one observed by us after 3 days (Jornayvaz *et al*. 2010), whereas other studies report universally beneficial effects on glucose metabolism resulting from KD feeding (Kennedy *et al*. 2007; Badman *et al*. 2009). Two recent studies looking at KD feeding in rodents for a time span of years complicate the picture. Roberts *et al*. (2017) report drastically impaired systemic glucose clearance, consistent with the findings of the present study and those of Jornayvaz *et al*. (2010), yet improved insulin sensitivity in aged mice on a live‐long KD, whereas Newman *et al*. (2017) do not report any functional data on glucose homeostasis. Our own studies and those of Jornayvaz *et al*. (2010) were performed in young mice, and so a plausible interpretation of these data is that impaired glucose tolerance is a direct metabolic effect of KD feeding, whereas life‐long low levels of glucose and insulin might counter the age‐dependent insulin resistance, which appears to involve a different mechanism than diet‐induced insulin resistance (Bapat *et al*. 2015).

Persistent low glucose and insulin plasma levels leading to a shift of lipid oxidation as the main fuel, as well as ketogenesis to preserve glucose, are key features that are shared by KD feeding and starvation. This is pertinent in light of the literature on starvation diabetes (Peters, 1945; Lundbaek, 1948), which suggests that 1–3 days of starvation or carbohydrate depletion is sufficient to impair systemic glucose clearance. It is possible that 3 days of carbohydrate depletion is sufficient to achieve the metabolic adaptation to KD and that the effects observed after longer duration reported by Jornayvaz *et al*. (2010) are not a result of impairments caused by long‐term KD but, instead, reflect the normal physiological adaptation to low carbohydrate intake. In the context of obesogenic HFD (typically containing ∼60% of calories from fat and 20% from carbohydrates), the impaired ability of insulin to reduce EGP after a few days of HFD feeding has been repeatedly observed in mice and men (Song *et al*. 2001; Brøns *et al*. 2009; Wiedemann *et al*. 2013). Moreover, elevated EGP is also known to be a key contributor of the elevated glucose levels occurring in T2D (Hundal *et al*. 2000). Our finding that, in both short‐term KD and HFD feeding, tissue glucose uptake is normal, is also in line with a recent report analysing the effect of obesogenic 60% HFD‐feeding for different time‐spans (Turner *et al*. 2013), which demonstrated that three days of HFD feeding caused insulin resistance specifically at the level of the liver. By contrast, impairments of muscle glucose uptake were only observed after 3 weeks and longer of HFD feeding. On the other hand, it has long been reported that low insulin levels themselves can cause peripheral insulin resistance in type 1 diabetes. However, this insulin resistance affects primarily the muscle (and not the liver), suggesting that this effect is unrelated to the causes of insulin resistance stemming from short‐term high‐fat diet consumption (DeFronzo *et al*. 1982a, *b*).

It is clear that, although there are important metabolic similarities between KD feeding and starvation, there are also substantial differences. Both starvation and dietary carbohydrate depletion lead to a switch of primary fuel from carbohydrate to lipid, as well as ketogenesis. However, starvation causes a pronounced reduction in energy expenditure and heat production (Cahill *et al*. 1966; Cahill, 1970; Jensen *et al*. 2013), whereas KD provides ample energy and reportedly causes either increased or unchanged energy expenditure (Jornayvaz *et al*. 2010; Paoli *et al*. 2013; Hall *et al*. 2016). The consequences of starvation resulting from reduced energy intake, rather than reduced carbohydrate intake, include increased autophagy and a reduced metabolic rate. Thus, it is possible that the symptoms of short‐term KD feeding in the present study, and the reported symptoms of starvation diabetes are caused by different mechanisms.

The significant correlation of RER and impaired glucose homeostasis suggests otherwise. In terms of the primary fuel for oxidation, our data show that animals on a regular chow diet primarily oxidize carbohydrates, whereas animals on a KD oxidize almost exclusively lipids and their RER does not move at all during fasting, with the HFD fed animals lying in between, oxidizing somewhat more lipids than carbohydrates. Remarkably, this fuel preference predicts the impairment to glucose homeostasis assessed by all three methods (GTT, ITT and glucose clamp). The severity of the impairment correlates linearly with the degree of lipid oxidation, with KD fed animals being most impaired and HFD fed animals lying in between. Importantly, the RER measurements during GTT and the glucose clamp studies reveal that the impairment does not occur at the level of the rate of glucose disappearance from plasma, tissue glucose uptake or the ability to oxidize glucose. In the fasted or carbohydrate‐depleted state, which is typically when rates of FFA oxidation are high in healthy individuals, increased hepatic glucose output is a vital necessity. Because the depletion of liver and muscle glycogen stores requires replenishment, it is plausible to propose that substantially elevated rates of FFA oxidation persisting for durations longer than 18–24 h is interpreted by part of the physiological homeostat as a signal for glucose depletion. This signal might result in a deliberate modulation of the ability of insulin to block endogenous glucose output to replenish muscle glycogen stores after periods of glucose paucity. Such a signal probably acts via the CNS, as reported previously (Girard, 2006), and is entirely consistent with the observed phenotype of starvation diabetes, which should more accurately be termed glucose depletion‐dependent impaired glucose tolerance.

Although animals on a HFD for 3 days are by no means diabetic, long‐term HFD feeding is the quintessential animal model for diet‐induced insulin resistance leading to T2D, and it is clear that, also in humans, the impact of an obesogenic diet occurs very long before overt T2D (Nathan, 2002; Boden *et al*. 2015). However, these initial causes and triggers are still incompletely understood. In terms of the long‐term progression of the disease, inflammation has been prominently invoked in the discussion of insulin resistance in the context of T2D. Dating back to a seminal study by Hotamisligil *et al*. (1993), a large body of evidence has convincingly demonstrated a causal link between inflammatory processes and peripheral insulin resistance, in particular in muscle and adipose tissue (Gregor & Hotamisligil, 2011). The current frontrunner for the first injury following a switch to obesogenic HFD is acute activation and inflammation of astrocytes and microglia, particularly in the ventromedial hypothalamus (Horvath *et al*. 2010; Thaler *et al*. 2011). However, a recent study in which inflammatory activation of microglia was blocked in the CNS demonstrated alterations in energy expenditure and intake, although no changes in glucose homeostasis were observed (Valdearcos *et al*. 2017). Moreover, a recent study analysing injury in the CNS resulting from the consumption of high‐fat, low carbohydrate or high‐fat, high carbohydrate diets only found damage following the high‐fat, high carbohydrate diets (Gao *et al*. 2017). Furthermore, a study examining the effects of inflammation on HFD induced insulin resistance over time reported a biphasic effect; short‐term effects were independent of inflammation, whereas long‐term effects responded well to various anti‐inflammatory interventions (Lee *et al*. 2011). These data lend credence to the interpretation that the early increase in hepatic glucose production following a HFD challenge is not caused by injury but, instead, represents a physiological response to drastically elevated fat intake and a shift in metabolic fuel preference.

Interestingly, in our studies, only the HFD animals showed signs of impaired glucose homeostasis when observed in the 6 h fasted state, even though the effect on hepatic glucose output under insulin was stronger in the KD fed group. The key difference between these two diets is that the obesogenic HFD provides significant amounts of calories from both fat and carbohydrate, whereas the non‐obesogenic KD provides calories almost exclusively from fat. It is relevant to note that a low carbohydrate KD does not cause obesity and that muscle insulin sensitivity appears to be entirely preserved in this model even after long periods of feeding, in marked contrast to obesogenic HFD (Jornayvaz *et al*. 2010; Garbow *et al*. 2011). These observations are readily explained assuming that persistently elevated lipid oxidation causes a signal to be transmitted to indicate glucose paucity and that the fat oxidation‐dependent blunting of the ability of insulin to suppress EGP is probably not linked to any damage and is reversible. This is a necessary adaptation in animals on a KD. However, in animals consuming large amounts of fat together with significant amounts of carbohydrate (a hallmark of the so‐called Western diet), the increased flux of glucose from endogenous production combines with the dietary glucose, leading to a progressive deterioration of systemic insulin sensitivity caused by the elevated insulin levels released from the pancreas to cope with elevated postprandial and fasting glucose levels.

## Additional information

### Competing interests

The authors declare that they have no competing interests.

### Author contributions

GG developed the hypothesis and project, performed the experiments, analysed data, and wrote the manuscript. MA performed the surgeries and assisted in the clamp studies. SW supervised and assisted in the clamp studies and analysed data. LS co‐performed the clamp and metabolic cage studies and analysed the data. CR performed the plasma analysis. DK analysed data and provided supervision. CW supervised the project, analysed data and wrote the manuscript. All authors approved the final version of the manuscript submitted for publication and agree to be accountable for all aspects of the work in ensuring that questions related to the accuracy or integrity of any part of the work are appropriately investigated and resolved. All persons designated as authors qualify for authorship, and all those who qualify for authorship are listed.

### Funding

Funding was provided by the Schweizer Nationalfonds (SNF).
